# The lateral parabrachial nucleus, but not the thalamus, mediates thermosensory pathways for behavioural thermoregulation

**DOI:** 10.1038/s41598-017-05327-8

**Published:** 2017-07-10

**Authors:** Takaki Yahiro, Naoya Kataoka, Yoshiko Nakamura, Kazuhiro Nakamura

**Affiliations:** 1 Department of Integrative Physiology, Nagoya University Graduate School of Medicine, Nagoya, 466-8550 Japan; 2 PRESTO, Japan Science and Technology Agency, Kawaguchi, Saitama, 332-0012 Japan

## Abstract

Thermoregulatory behaviour, such as migration to a comfortable thermal environment, is a representative innate animal behaviour and facilitates effective autonomic regulation of body temperature with a reduced cost of resources. Here we determine the central thermosensory ascending pathway that transmits information on environmental temperature from cutaneous thermoreceptors to elicit thermoregulatory behaviour. To examine the contribution of the spinothalamocortical pathway, which is known to mediate thermosensory transmission for perception of skin temperature, we lesioned thalamic regions mediating this pathway in rats. Thalamic-lesioned rats showed compromised electroencephalographic responses in the primary somatosensory cortex to changes in skin temperature, indicating functional ablation of the spinothalamocortical pathway. However, these lesioned rats subjected to a two-floor innocuous thermal plate preference test displayed intact heat- and cold-avoidance thermoregulatory behaviours. We then examined the involvement of the lateral parabrachial nucleus (LPB), which mediates cutaneous thermosensory signaling to the thermoregulatory center for autonomic thermoregulation. Inactivation of neurons in the LPB eliminated both heat- and cold-avoidance thermoregulatory behaviours and ablated heat defense. These results demonstrate that the LPB, but not the thalamus, mediates the cutaneous thermosensory neural signaling required for behavioural thermoregulation, contributing to understanding of the central circuit that generates thermal comfort and discomfort underlying thermoregulatory behaviours.

## Introduction

Regulation of body temperature is one of the most critical homeostatic functions that are governed by the central nervous system. Thermoregulatory measures inherent in homeothermic animals including humans are classified into involuntary autonomous (i.e., autonomic and shivering) responses and voluntary behavioural responses. Understanding the central circuit mechanisms that control autonomous thermoregulatory responses, such as shivering in skeletal muscles, non-shivering thermogenesis in brown adipose tissue and vasomotion in skin blood vessels, has markedly progressed^1, 2^. On the other hand, the central circuits underlying behavioural thermoregulation have been poorly understood. By employing thermoregulatory behaviour, represented by cool- and warm-seeking behaviours in hot and cold environments, respectively, animals choose an optimal thermal environment so that their autonomous thermoregulatory mechanisms can efficiently maintain body core temperature. This instinctive behaviour is particularly important when animals are in severe thermal environments where autonomous thermoregulatory mechanisms have a limited effect. Thermoregulatory behaviour can also reduce the energy cost of autonomous thermogenesis in cold environments and the risk of dehydration in hot environments^3^.

For the defense of body core temperature from environmental thermal challenges, changes in environmental temperature are detected by cutaneous thermoreceptors located on endings of primary somatosensory nerve fibres and the thermosensory signals are transmitted through the spinal dorsal horn to the brain to initiate appropriate thermoregulatory behaviours before their body core temperature is impacted by the thermal challenges. However, the afferent neural pathways that transmit the cutaneous thermosensory signals from the spinal dorsal horn for behavioural thermoregulation are unknown.

Thermoregulatory behaviour is closely related to thermal comfort, as it has been defined as “an attempt to avoid what humans call thermal discomfort or displeasure and to obtain thermal pleasure”^4^. Consistently, behavioural thermoregulation has been proposed to involve several forebrain regions potentially related to emotional behaviour, such as the insular, cingulate and somatosensory cortices and the amygdala^5^, which are activated in response to cutaneous innocuous thermal stimuli^6–8^. The activation of the cortical regions by thermosensory information from the skin gives rise to the idea that the thermosensory afferent signaling for behavioural thermoregulation is mediated by the spinothalamocortical pathway, in which cutaneous thermosensory signals are transmitted from the spinal dorsal horn to the thalamus and then relayed to the primary somatosensory cortex^9, 10^. This pathway is responsible for perception and discrimination of skin temperature^9, 10^, but does not function for cool-sensory signaling to drive sympathetic thermogenesis in brown adipose tissue^11^.

On the other hand, we have identified another thermosensory pathway, in which cutaneous thermosensory signals are transmitted from the dorsal horn to the lateral parabrachial nucleus (LPB) and then to the preoptic area (POA) of the hypothalamus^11, 12^. The spinal–LPB–POA pathway transmits cutaneous warm- and cool-sensory signals separately to the thermoregulatory center in the POA to drive autonomous thermoregulatory responses to environmental thermal challenges, including shivering and non-shivering thermogenesis and cutaneous vasomotion^11, 12^. However, involvement of the thermoregulatory center in the POA in behavioral thermoregulation is controversial^13^.

For understanding the central circuit mechanism of behavioural thermoregulation, therefore, it is essential to determine the afferent neural pathways that transmit cutaneous thermosensory signals to elicit thermoregulatory behaviour. Identification of the thermosensory pathways for behavioural thermoregulation will also provide important information to understand the central circuits generating thermal comfort and discomfort, which are likely emotional motivations for thermoregulatory behaviour. In the present study, we ablated the spinothalamocortical pathway or LPB-mediated pathways of rats by pharmacological lesion or inactivation of neurons, and examined the effect of this ablation on cold- and heat-avoidance thermoregulatory behaviours measured by a two-floor thermal plate preference test.

## Results

### Cold- and heat-avoidance behaviours do not require thalamic relay

**Figure 1:**
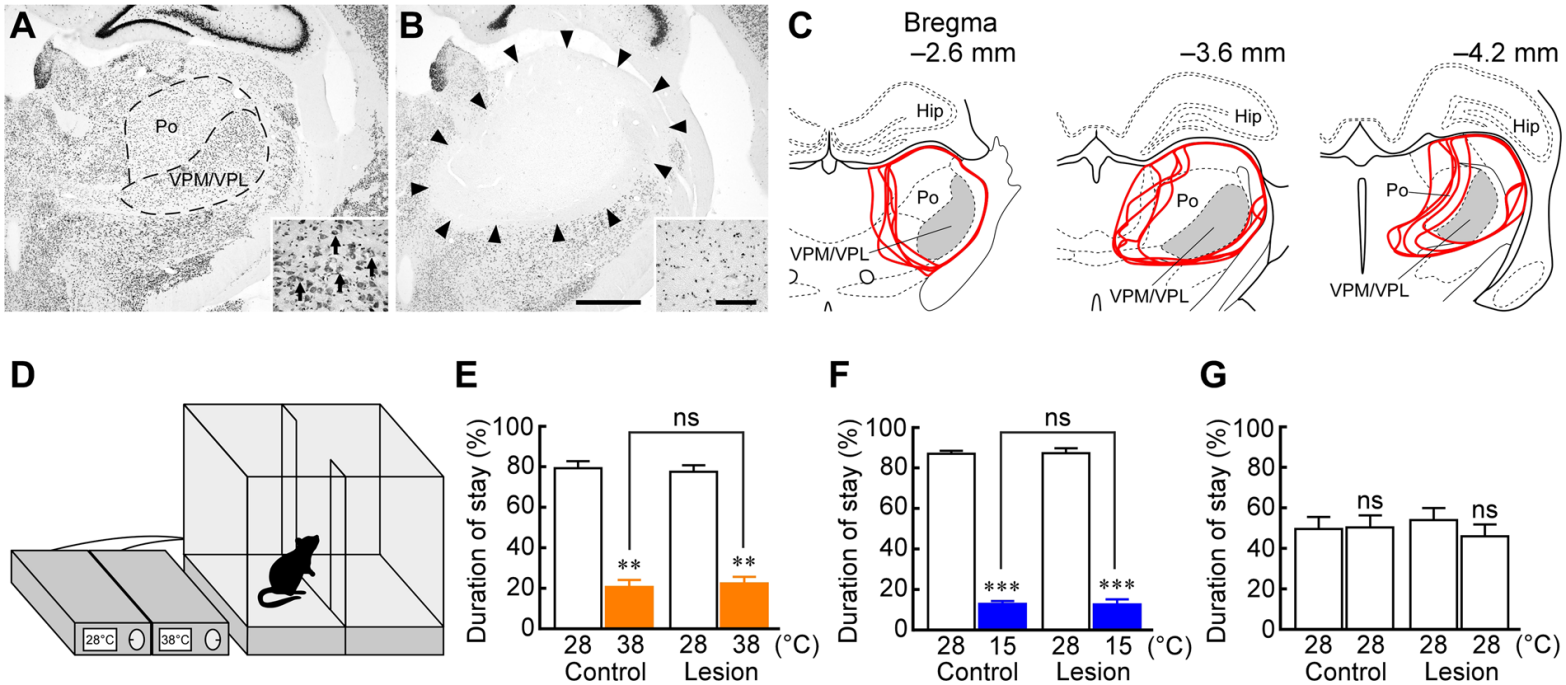
Thalamic lesions have no effect on heat- or cold-avoidance thermoregulatory behaviour. (**A** and **B**) NeuN immunohistochemistry and cresyl violet staining (insets) in the thalamus of saline-injected control (**A**) and thalamic-lesioned (**B**) rats. Ibotenate injections eliminated neurons in an area including the VPM/VPL and posterior thalamic nuclear group (Po) ((**B**) delineated by arrowheads) as compared with the control (**A**). One side of the bilateral injection sites is shown. Cresyl violet staining in the insets shows that large, neuron-like cells are found in the VPM/VPL of control rats ((**A**) arrows), but not of lesioned rats, which contained gliosis (**B**). Scale bars, 1 mm (**A** and **B**), 100 µm (insets). (**C**) Thalamic areas lesioned with ibotenate injections. Lesioned areas from all the animals (*n* = 5) are delineated (red) and overlaid at three rostrocaudal levels. Gray areas indicate the VPM/VPL. The right side of the symmetric bilateral lesions is shown. Hip, hippocampus. (**D**) Thermal plates and temperature controllers used for thermal plate preference tests. The actual enclosure was not transparent. (**E**–**G**) Thermal plate preference tests of saline-injected control and thalamic-lesioned rats (*n* = 5 each) on the plates set at 28 °C *vs* 38 °C (**E**), 28 °C *vs* 15 °C (**F**) or 28 °C *vs* 28 °C (**G**). Duration of stay on each plate is expressed as % of the total test period (20 min). **P < 0.01; ***P < 0.001, compared with the duration of stay on the 28 °C plate (two-tailed paired *t*-test). Comparisons of the duration of stay on the 38 °C plate (**E**) and 15 °C plate (**F**) between the control and lesioned groups were performed with two-tailed unpaired *t*-tests. ns, not significant.

To examine whether the spinothalamocortical pathway contributes to thermosensory afferent transmission required for thermoregulatory behaviour, we bilaterally lesioned the ventral posteromedial and ventral posterolateral thalamic nuclei (VPM/VPL), thalamic regions receiving a majority of thermal somatosensory spinothalamic projections in rats^14, 15^. Injections of ibotenate into the VPM/VPL lesioned most of these thalamic regions as observed by postmortem histology; the lesioned areas were clearly identified as they contained no immunoreactivity for NeuN, a neuronal marker, and were filled with small, glia-like cells stained with cresyl violet (Fig. 1A–C). The thalamic-lesioned rats survived without exhibiting any obvious symptoms, comparable to saline-injected control rats.

The saline-injected and thalamic-lesioned rats were subjected to two-floor innocuous thermal plate preference tests. As shown in Fig. 1D, a rat moved freely on two thermal floor plates, one of which was set at 28 °C, which is in the thermoneutral zone of environmental temperature for laboratory rats^16^, while the other was set at 15 (cool), 28 (neutral) or 38 °C (warm). Each behavioural test was conducted for 20 min under room temperature of 25 °C. Saline-injected rats preferred staying on a 28 °C plate to a 38 °C or 15 °C plate (Fig. 1E and F), showing typical heat- and cold-avoidance thermoregulatory behaviours, respectively. Of note, thalamic-lesioned rats also exhibited obvious heat- and cold-avoidance behaviours comparable to control rats (Fig. 1E and F). Temperature assignment to the two plates was random, and when they were both set at 28 °C, either animal group almost equally stayed on both sides (Fig. 1G), confirming that the plate selection behaviours observed were not due to their temperature-independent preference of either specific side and also that the lesioned animals had no impairment of their locomotor function.

**Figure 2:**
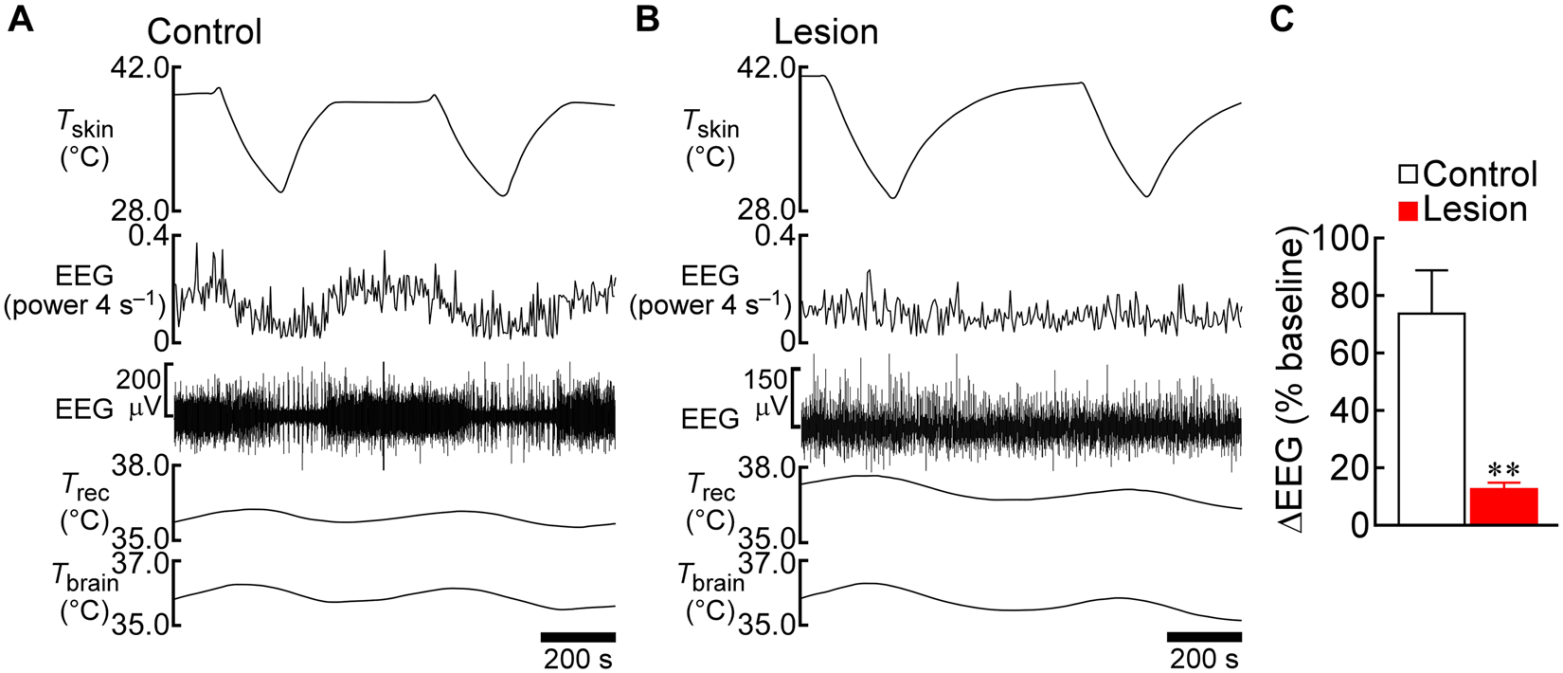
Thalamic lesions eliminate somatosensory cortical EEG responses to changes in skin temperature. (**A** and **B**) Skin cooling-evoked changes in EEG recorded from the primary somatosensory cortex of the control (**A**) and thalamic-lesioned (**B**) rats from Fig. 1. *T*_brain_, brain temperature; *T*_rec_, rectal temperature; *T*_skin_, skin temperature. (**C**) Group data showing the effect of thalamic lesion on the skin cooling-evoked changes in EEG (*n* = 5 each). Skin cooling-evoked changes (absolute values) in the EEG “power 4 s^−1^” traces (**A** and **B**) from the pre-cooling baseline to average value during the 30-sec period immediately before the end of skin cooling are expressed as % of the pre-cooling baseline value (averaged from 2 cooling episodes in each rat). **P < 0.01 (two-tailed unpaired *t*-test).

To test whether the spinothalamocortical pathway was functionally ablated in the thalamic-lesioned rats, the animals were subjected to electroencephalography (EEG) from the primary somatosensory cortex after the thermal plate preference tests. The trunk skin was repeatedly cooled and rewarmed under anesthesia and the effect of the cutaneous thermal stimuli on EEG activity was examined. In the saline-injected control rats, skin cooling consistently changed EEG activity. Four of the five control rats exhibited decreases in EEG power in response to skin cooling and increases in response to rewarming (Fig. 2A), whereas the rest of the control rats showed opposite changes in EEG activity as seen in our previous study^11^. On the other hand, the changes in EEG activity in response to cutaneous thermal stimuli were eliminated in the thalamic-lesioned rats (Fig. 2B and C). These results indicate that the spinothalamocortical pathway was functionally ablated in the thalamic-lesioned rats, but nonetheless, they were able to display heat- and cold-avoidance thermoregulatory behaviours.

### Cold- and heat-avoidance behaviours require activation of LPB neurons

**Figure 3:**
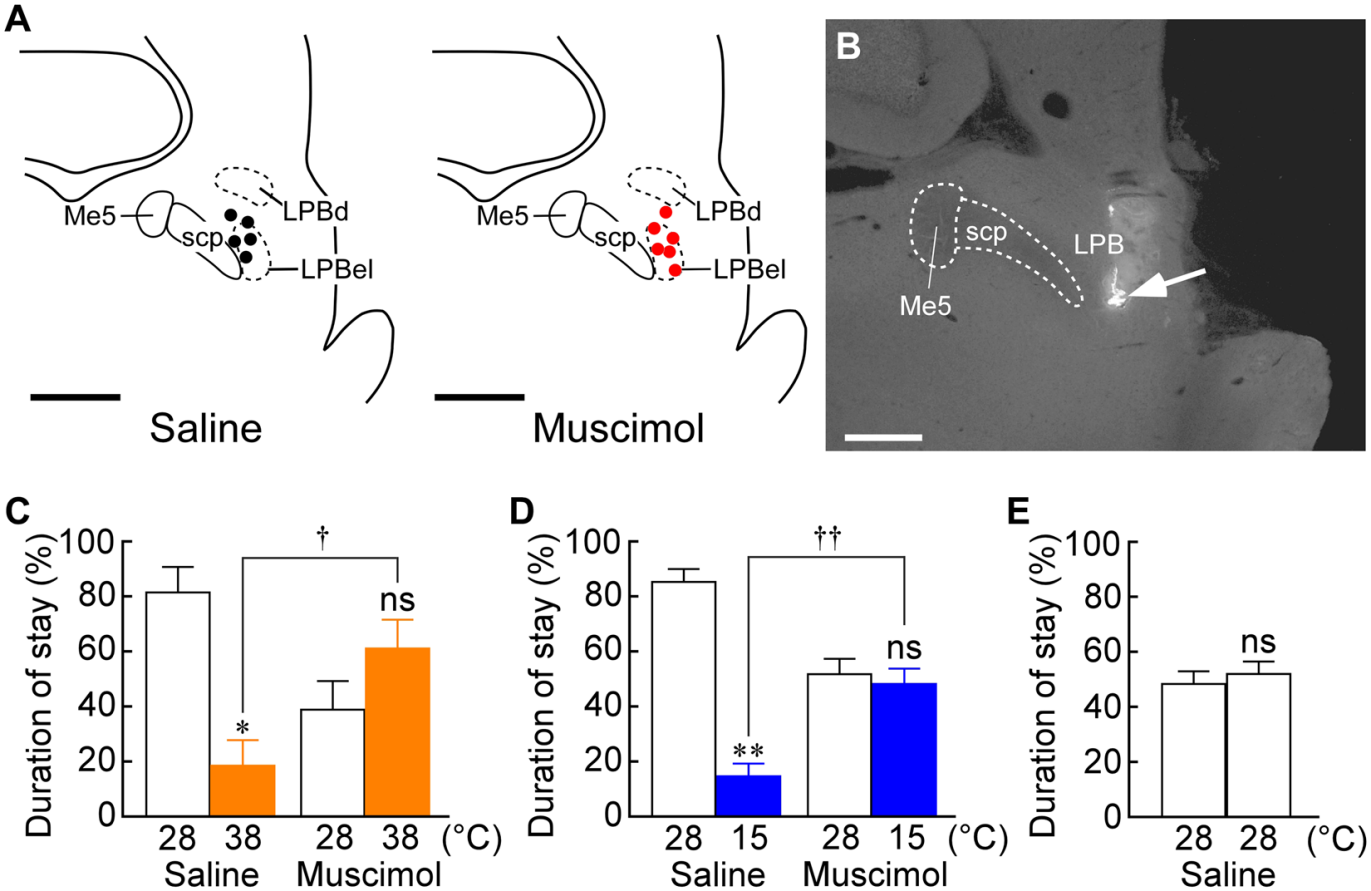
Muscimol injections into the LPB eliminate heat- and cold-avoidance thermoregulatory behaviours. (**A**) Location of the sites of saline (black circles) and muscimol (red circles) nanoinjections in the LPB. One side of the symmetric bilateral injections is shown. Scale bars, 1 mm. Me5, mesencephalic trigeminal nucleus; scp, superior cerebellar peduncle. (**B**) A representative view of an injection site in the LPB (arrow). Scale bar, 0.5 mm. (**C**–**E**) Thermal plate preference tests of rats following bilateral nanoinjections into the LPB with saline (*n* = 5) or muscimol (*n* = 6) on the plates set at 28 °C *vs* 38 °C (**C**), 28 °C *vs* 15 °C (**D**) or 28 °C *vs* 28 °C (**E**). Duration of stay on each plate is expressed as % of the total test period (20 min). *P < 0.05; **P < 0.01, compared with the duration of stay on the 28 °C plate (two-tailed paired *t*-test). ^†^ *P* < 0.05; ^††^ *P* < 0.01 (two-tailed unpaired *t*-test).

To examine whether the LPB, another potential brain site for thermosensory signaling, is involved in behavioural thermoregulation, we tested the effect of a blockade of LPB-mediated thermosensory pathways on the heat- and cold-avoidance thermoregulatory behaviours. Because bilateral lesions of the LPB with ibotenate injections were lethal to rats in our pilot experiments, we chose to inactivate LPB neurons by nanoinjecting muscimol, a GABA_A_ receptor agonist widely used as a neuronal inhibitor, bilaterally into the LPB of free-moving rats through preimplanted intracranial cannulae (Fig. 3A and B). Similar to the rats that received saline injections into the VPM/VPL, those that received bilateral control nanoinjections into the LPB with saline exhibited strong preference of a 28 °C plate to a 38 °C or 15 °C plate (Fig. 3C and D). In contrast, inactivation of neurons in the LPB with muscimol nanoinjections eliminated both heat- and cold-avoidance behaviours (Fig. 3C and D). Muscimol-injected rats moved back and forth on the plates without showing any sign of impaired motor functions. Saline-injected rats did not prefer a specific side when both plates were set at 28 °C (Fig. 3E).

**Figure 4:**
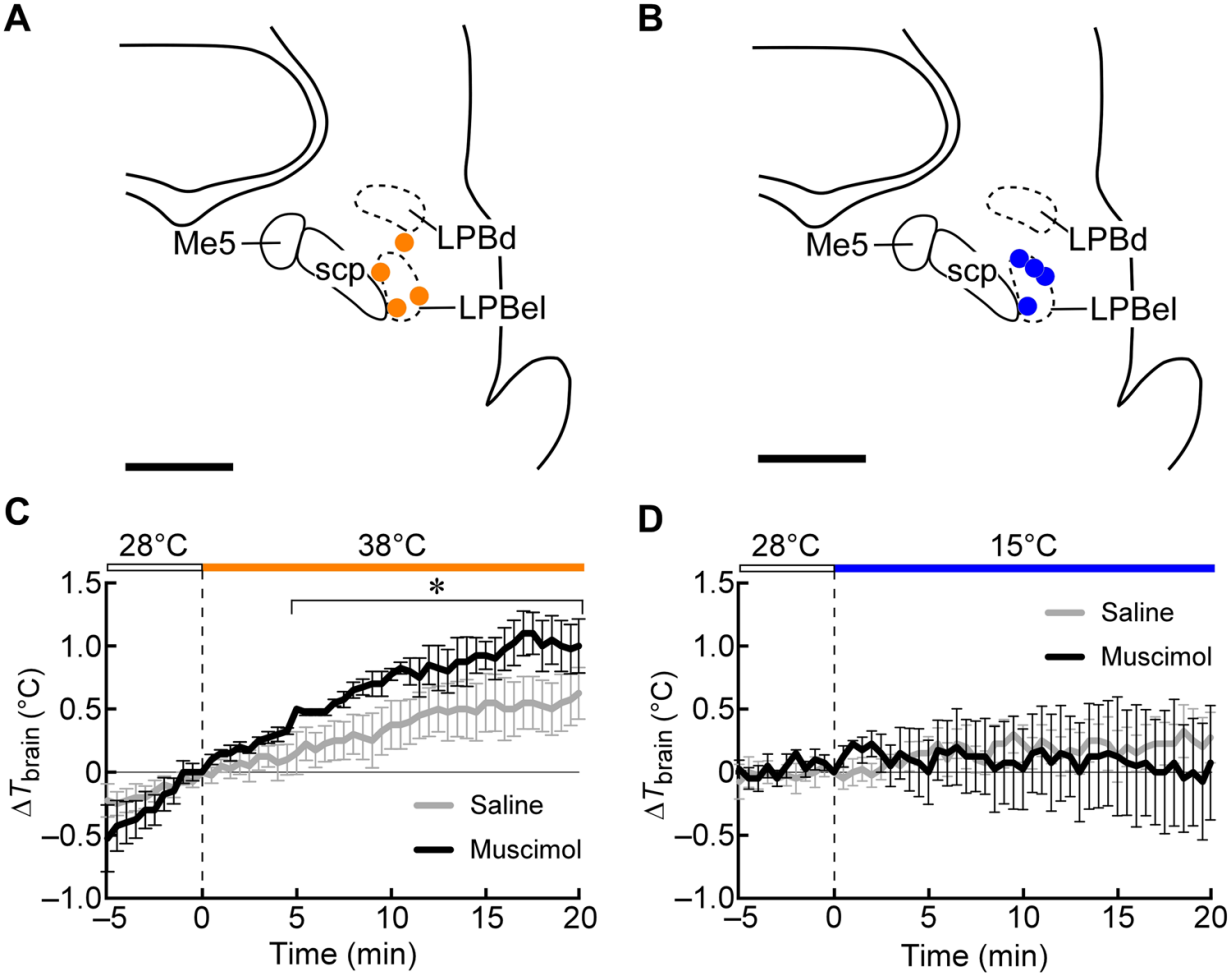
Effects of muscimol injections into the LPB on brain temperature of rats on a warm or cool plate. (**A** and **B**) Location of the sites of saline and muscimol nanoinjections in the LPB of rats whose *T*_brain_ was recorded on a 38 °C ((**A**) orange circles) or 15 °C ((**B**) blue circles) plate. One side of the symmetric bilateral injections is shown. Scale bars, 1 mm. (**C** and **D**) Changes in *T*_brain_ of rats transferred from a 28 °C plate to a 38 °C (**C**) or 15 °C (**D**) plate (*n* = 4 each) following bilateral nanoinjections of saline or muscimol into the LPB in a room air-conditioned at 25 °C. The nanoinjections were made 10 min before the transfer to the warm or cool plate. Each rat received nanoinjections at the same LPB sites with saline and muscimol with an interval of 1 week to examine their effects on *T*_brain_ in the same animals under the same plate temperature setting. *P < 0.05, Bonferroni multiple comparisons test following a two-way repeated measures ANOVA.

The present results show that inactivation of LPB neurons eliminates behavioural thermoregulation. Blockade of neurotransmission in the LPB also diminishes feedforward autonomous thermoregulatory responses to skin cooling and warming in anesthetized rats^11, 12^. Therefore, inactivation of LPB neurons likely leads to failure of the defense of body core temperature in hot and cold environments; however, it has not been demonstrated in free-moving animals. To examine the importance of circuit mechanisms mediated by LPB neurons in the defense of thermal homeostasis, we compared the effects of saline and muscimol nanoinjections into the LPB on brain temperature of rats placed on the warm or cool plate used in the thermal plate preference tests. Rats following bilateral nanoinjections into the LPB with either saline or muscimol (Fig. 4A and B) showed comparable brain temperature on a 28 °C plate under room temperature of 25 °C (saline, 37.1 ± 0.3 °C *vs* muscimol, 37.4 ± 0.2 °C immediately before transferred to a 38 °C plate, *n* = 4, *P* = 0.56, two-tailed paired *t*-test; saline, 37.1 ± 0.2 °C *vs* muscimol, 37.0 ± 0.5 °C immediately before transferred to a 15 °C plate, *n* = 4, *P* = 0.74). Following transferred to a 38 °C plate (Fig. 4C), both saline-injected and muscimol-injected rats showed gradual increases in brain temperature, but muscimol-injected rats exhibited a significantly larger increase in brain temperature, which peaked in a hyperthermic range (38.5 ± 0.3 °C) within 20 min (time, *F*_40, 120_ = 9.527, *P* < 0.001; injectate, *F*_1, 3_ = 9.214, *P* = 0.056; time × injectate, *F*_40, 120_ = 3.755, *P* < 0.001, two-way repeated measures ANOVA), whereas saline-injected rats maintained their brain temperature below 38.0 °C. This result shows that the rats whose LPB neurons were inactivated failed to defend their body core temperature on the warm plate, leading to the development of hyperthermia. Following transferred to a 15 °C plate (Fig. 4D), in contrast, both saline-injected and muscimol-injected rats kept their brain temperature relatively constant and muscimol injections into the LPB had no significant effect on the changes in the brain temperature for the 20-min test period (time, *F*_40, 120_ = 0.3183, *P* > 0.999; injectate, *F*_1, 3_ = 0.0431, *P* = 0.849; time × injectate, *F*_40, 120_ = 0.581, *P* = 0.975, two-way repeated measures ANOVA).

## Discussion

It has been speculated that the thalamocortical pathway mediates the afferent transmission of cutaneous thermosensory signaling for behavioural thermoregulation^17^. However, the present study clearly demonstrates that the thalamocortical thermosensory transmission is not required for eliciting heat- or cold-avoidance behaviour, while the LPB is a brain site that plays an essential role in the central circuit for these thermoregulatory behaviours. The LPB receives numerous projections from the dorsal horn^18–20^, which include axonal collaterals from spinothalamic and trigeminothalamic projections^21, 22^. The LPB contains two separate populations of neurons responsive to either cutaneous warm- or cool-sensory input; they are activated by exposure of animals to warm or cool ambient temperature or by direct warming or cooling of the trunk skin with a water jacket^11, 12, 23^. Inactivation of LPB neurons eliminates autonomous thermoregulatory responses to skin warming and cooling, but does not inhibit sympathetic thermogenesis evoked by an action of a pyrogenic mediator in the thermoregulatory center, POA^11, 12^. Therefore, the LPB neurons, which are activated by cutaneous thermosensory inputs from spinal and medullary secondary sensory neurons, mediate ascending transmission of environmental thermal information required for autonomous thermoregulation, but not descending transmission of command signals from the POA to thermoregulatory effectors^1, 2, 11, 12^. However, contribution of LPB neurons to thermosensory signaling for behavioural thermoregulation has been unknown.

In the present study, control rats with saline injections preferred a plate of thermoneutral temperature to a warm or cool plate, obviously displaying thermoregulatory behaviour to avoid the excessive heat or cold, respectively. We found that these heat- and cold-avoidance thermoregulatory behaviours were eliminated by inactivation of LPB neurons. This result indicates that activation of LPB neurons is required for eliciting thermoregulatory behaviours to avoid excessive heat and cold. Together with our previous finding that the LPB mediates cutaneous thermosensory signaling^11, 12^, the present finding raises the view that the LPB-mediated thermosensory signaling constitutes the afferent arm of both autonomous and behavioural thermoregulatory circuit systems.

Demonstrating the importance of the LPB-mediated neural signaling, our brain temperature recording showed that rats whose LPB neurons were inactivated failed to defend their body core (brain) temperature on a warm plate and exhibited hyperthermia. Similar hyperthermia was likely developed in the rats with muscimol injections into the LPB during the thermal plate preference test with the 28 °C *vs* 38 °C setting. Nonetheless, they chose to stay on the warm plate for more than half the test period (Fig. 3C), indicating that inactivation of LPB neurons causes a strong defect in heat-avoidance behaviour. In contrast, rats with inactivated LPB neurons were able to maintain their body temperature on a 15 °C plate for 20 min. This result might suggest that the LPB-mediated neural signaling makes a smaller contribution to cold defense. Even when cooling-induced shivering and brown adipose tissue thermogenesis are inhibited by inactivation of LPB neurons^11^, basal heat production from general physiological activities within the body, such as cellular metabolism and muscle movements, could support the defense of body core temperature in a cool environment if it is not severe cold. The present recordings of brain temperature were performed under a room temperature of 25 °C and therefore, a dominant area of the rat skin was not cooled. When the whole body is exposed to cool air (16–17 °C), normal rats can maintain their body core temperature, but rats with bilateral electrolytic lesions in the LPB exhibit a reduction of body core temperature^24^, although this lesion technique destroys both cells and passing fibers in contrast to the cell body-selective lesion with ibotenate in the present study. Therefore, the autonomous and behavioural thermoregulatory responses elicited by the LPB-mediated afferent transmission of cutaneous thermosensory information seem more important for the defense of thermal homeostasis in hot environments than in cold environments, but they are still required to survive severe cold.

The LPB neurons activated by cutaneous warm-sensory inputs are localized in the dorsal part of the LPB (LPBd), while those activated by cutaneous cool-sensory inputs are in the external lateral part of the LPB (LPBel)^11, 12^. Although intracranial cannulae for muscimol injections in the present study were mostly placed around the LPBel, the muscimol injections eliminated both heat- and cold-avoidance behaviours. This is likely because the injectate diffused into the LPBd, which is located in close proximity to the LPBel, before starting the thermal plate preference test. Both cutaneous warm-sensory and cool-sensory populations of LPB neurons provide direct glutamatergic projections to the median part of the POA, median preoptic nucleus^11, 12^. Autonomic heat-defense and cold-defense responses elicited by stimulation of LPBd and LPBel neurons, respectively, were both eliminated by blockade of glutamate receptors in the median preoptic nucleus^11, 12^. Inactivation of neurons in the median preoptic nucleus eliminates skin cooling-induced thermogenesis in brown adipose tissue^25^ and blockade of glutamate receptors in the median preoptic nucleus eliminates skin warming-induced cutaneous vasodilation^12^. These findings indicate that the pathway from the LPB to the POA transmits cutaneous thermosensory signals to the thermoregulatory center that are required for eliciting autonomous thermoregulatory responses to environmental thermal changes.

However, the contribution of the POA to behavioural thermoregulation has been controversial. Electrolytic or thermal lesion of the POA, which strongly attenuates the ability of autonomous thermoregulation, does not affect operant thermoregulatory behaviours, such as conditioned lever-pressing to increase or decrease ambient temperature or to escape from an extreme thermal environment^26, 27^. POA-lesioned rats exhibit severe hypothermia in a cold environment due to ablated autonomous thermoregulation, but can maintain their body core temperature if operant thermoregulation is allowed^28^. Furthermore, electrolytic lesion of the POA does not affect warm- or cold-seeking behaviour^29^. On the other hand, there are reports that support a role of the POA in behavioural thermoregulation. Rats with pharmacological (NMDA-induced) lesion of the medial POA prefer warmer ambient temperature than control rats^30^. A recent study has shown that optogenetic stimulation of a population of POA neurons that can be physiologically activated in response to skin warming induces cold-seeking behaviour^31^. Therefore, cutaneous thermosensory signals transmitted through the LPB to the POA likely alter activities of thermoregulatory POA neurons to induce thermoregulatory behaviours as well as autonomous thermoregulatory responses.

Thermoregulatory behaviour has been regarded as an instinct behaviour underlain by thermal comfort and discomfort and therefore, thought to involve several cortical regions related to emotion and perhaps thermal comfort, such as the insular, cingulate and somatosensory cortices and the amygdala^5^. Functional magnetic resonance imaging studies in humans have shown that these cortical structures are activated by cutaneous innocuous thermal stimuli^6–8^. Therefore, the cortical processing of thermosensory information, which could contribute to the generation of thermal comfort and discomfort leading to thermoregulatory behaviour, seems to be initiated by the cutaneous thermosensory inputs to the primary somatosensory cortex through the spinothalamocortical pathway^9, 10^. In our present study, however, functional ablation of the thalamocortical thermosensory transmission had no effect on cold- or heat-avoidance thermoregulatory behaviour. Therefore, ascending thermosensory signaling through the spinothalamocortical pathway contributes to perception and discrimination of skin temperature, but not to the thermal information processing underlying thermoregulatory behaviour.

The present determination of the LPB as a brain site that mediates thermosensory signaling for behavioural thermoregulation would trigger new studies for the circuit mechanisms that generate thermal comfort and discomfort underlying this innate behaviour. The LPB mediates pain transmission to the amygdala through a direct projection to create fear memory leading to aversive behaviour^32, 33^. This finding gives rise to the hypothesis that innocuous thermosensory signals are also transmitted from the LPB to the amygdala for the generation of thermal comfort and discomfort. However, the LPB neurons mediating cutaneous thermosensory signaling do not seem to directly project to the amygdala. Most LPB neurons express either transcription factor FoxP2 or Lmx1b and these two neuronal populations show segregated distribution patterns^34^. The LPBel neurons activated by cool-sensory inputs and the LPBd neurons activated by warm-sensory inputs both express FoxP2^35^, whereas LPB neurons projecting to the central nucleus of the amygdala do not express FoxP2, but are distributed in a subregion where Lmx1b-expressing neurons are predominantly distributed^34, 36^.

It may be worthwhile to examine the possibility that POA neurons that are stimulated by LPB-mediated thermosensory signaling mediate the generation of thermal comfort and discomfort as a part of their thermoregulatory functions. POA neurons that can be activated by skin warming show a curious projection pattern in the forebrain: projecting to the bed nucleus of the stria terminalis, lateral septal nucleus, paraventricular thalamic nucleus and medial habenular nucleus^31^. Thermal preference tests combined with selective stimulation of POA-derived nerve endings in one of these projection sites using optogenetics or chemogenetics could determine the forebrain neurotransmission from the POA that mediates thermoregulatory behaviour and potentially thermal comfort and discomfort underlying the behavioural responses. Tracing the LPB–POA-mediated pathway may lead to elucidation of the central circuit mechanisms that generate emotional drives of innate behaviours to defend homeostasis from a variety of environmental stressors including thermal challenges.

## Methods

### Animals

Twenty-nine male Wistar rats (240–400 g) contributed to the present study. The animals were housed with ad libitum access to food and water in a room air-conditioned at 24 ± 2 °C with a standard 12 h light/dark cycle. All procedures conform to the guidelines of animal care by the Division of Experimental Animals, Nagoya University Graduate School of Medicine and to the regulations detailed in the National Institutes of Health Guide for the Care and Use of Laboratory Animals, and were approved by the Nagoya University Animal Experiment Committee (approval No. 28089).

### Surgery

Rats were deeply anesthetized with chloral hydrate (280 mg kg^−1^, i.p. injection with 7% solution). Adequacy of anesthesia was carefully verified during surgery by the absence of a hindlimb withdrawal to foot pinch and/or by the absence of eye blink response to gentle touch of the cornea. With the adequate level of anesthesia, no animal exhibited any sign of pain or discomfort during surgery or postoperative recovery. To ablate the spinothalamocortical pathway^11^, the animals were positioned in a stereotaxic apparatus and received bilateral injections of 50 mM ibotenate or saline (50 nl per site) into 6 sites per side throughout the VPM/VPL (3.0–3.5 mm caudal to bregma). The ibotenate injections extensively lesioned the VPM/VPL in the rostrocaudal range of 2.3–4.6 mm caudal to bregma (Fig. 1C).

For experiments to inactivate neurons in the LPB, rats were positioned in a stereotaxic apparatus under anesthesia and sterile guide cannulae (ID = 0.39 mm, OD = 0.71 mm; C313G; Plastic One, Roanoke, VA, USA) were perpendicularly inserted to target the LPB bilaterally (coordinates: 9.0 mm caudal to bregma, 2.6 mm lateral to the midline and 5.7 mm ventral to the brain surface). The guide cannulae were then anchored with dental cement to stainless steel screws attached to the skull. Dummy cannulae cut to the exact length of the guide cannulae were inserted into the guide cannulae to avoid clogging. Internal cannulae for injection (see below) with the thickness to fit the guide cannulae were cut to be long enough to allow the injector tip to protrude 1.0 mm below the tip of the guide cannulae. In some rats, an additional guide cannula (ID = 0.24 mm, OD = 0.46 mm; C315G; Plastic One) was implanted to target the basal forebrain (2.2 mm anterior to bregma, 1.3 mm left to the midline, 7.0–7.5 mm ventral to the brain surface) with a thermocouple to monitor brain temperature (see below).

After the surgery, the incisions in the skin were closed with suture or wound clips and the wounds were treated with iodine. The animals were given an injection of an ampicillin sodium solution (0.2 ml, 125 mg ml^−1^) into femoral muscles to avoid infection and housed individually for >1 week to recover from the surgery under regular health check until used for thermal plate preference tests.

### Thermal plate preference test

This test was conducted in a room air-conditioned at 25 ± 0.5 °C between 9:00 and 15:00. Two thermal plates (19 cm × 30 cm) were placed side by side, with a black Plexiglas enclosure (40 cm tall; see Fig. 1D). Each thermal plate contained Peltier devices to keep the surface temperature uniform across the entire plate. Temperature of each plate was regulated to within ±0.1 °C of the set temperature by a controller (Intercross, Tokyo, Japan). Plexiglas barriers separated the adjoining thermal plates, but a rat was allowed to pass freely between the two sides through the opening between the barriers (Fig. 1D). One plate was set at 28 °C and the other was set at 15, 28 or 38 °C. The temperature assignment to the two plates was not side-fixed. Immediately after a rat was placed on the plates, time of stay on each plate was recorded for 20 min. Duration of stay on each plate was calculated as percentage of the 20-min test period. The combinations of the plate temperatures used in the present study were determined in our pilot experiments, in which rats were tested with various combinations of innocuous plate temperatures and found that they consistently preferred the 28 °C plate under either 28 °C *vs* 15 °C or 28 °C *vs* 38 °C setting, exhibiting cold- and heat-avoidance thermoregulatory behaviours, respectively.

In thermal plate preference tests using the rats that received saline or ibotenate injections into the VPM/VPL (Fig. 1), each rat was tested with all the three plate temperature combinations (i.e., 28 °C *vs* 15 °C, 28 °C *vs* 28 °C and 28 °C *vs* 38 °C) on separate days. On each day, a 20-min test with one of the temperature combinations was repeated twice with an interval of ~1 hr (the rats returned to their home cages during the interval), and this repeated test session was performed on two separate days for each temperature combination (i.e, four 20-min tests performed for each temperature combination). Therefore, each rat was tested with the three temperature combinations in a random order for 6 separate days in total. The duration of stay (%) on each plate obtained from the four 20-min tests for each temperature setting was averaged for each animal (Fig. 1E–G). After the completion of all the thermal plate preference tests, the rats were subjected to EEG recording as described below.

In thermal plate preference tests using the rats that had the intracranial cannulation (Fig. 3), they received bilateral nanoinjections of either saline or muscimol into the LPB before thermal plate preference tests. An internal cannula was connected to a Teflon tubing and the inside of the cannula and tubing was filled with pyrogen-free saline or 2 mM muscimol (dissolved in saline). A Hamilton syringe (10 µl) filled with mineral oil was connected to the other end of the Teflon tubing. The dummy cannulae that had been inserted into the guide cannulae were gently removed and the internal cannula was inserted into one of the guide cannulae. Then, the saline or muscimol solution (50 nl/side) was slowly ejected through the cannula using a manually operated syringe manipulator (Narishige, Tokyo, Japan). Immediately after an injection on one side, another injection was made on the other side. The injection volume was visually confirmed by the movement of the aqua–oil interface along the Teflon tubing, which had been graduated. Five minutes after the completion of the bilateral nanoinjections into the LPB, the rats were repeatedly subjected to 20-min thermal plate preference tests by turns with plate temperature settings of 28 °C *vs* 15 °C and 28 °C *vs* 38 °C (and also 28 °C *vs* 28 °C for saline-injected rats) with intervals of >10 min until all the temperature settings were tested twice each. The initial plate temperature setting in this test session was randomly assigned for each rat. Although the test session following muscimol injections into the LPB lasted approximately 120 min, muscimol nanoinjected into the brain parenchyma has been confirmed to maintain its neuronal inhibitory effect for >120 min in *in vivo* physiological experiments using rats^37^. The duration of stay (%) on each plate obtained from the two 20-min preference tests for each temperature setting was averaged for each animal (Fig. 3C–E).

In the experiments to examine the effect of muscimol injections into the LPB on brain temperature (Fig. 4), a needle-type copper-constantan thermocouple (0.33 mm diameter; Physitemp) was inserted into the basal forebrain through the pre-implanted guide cannula, and connected to a thermocouple meter to monitor brain temperature. The rats then received bilateral nanoinjections into the LPB with a random selection of saline or muscimol. After the injections, the rats were placed on a 28 °C plate for 10 min and then, transferred to a 38 °C or 15 °C plate to stay there for 20 min. Their brain temperature was recorded every 30 sec. One week after this test, the same rats received bilateral injections of the other solution into the LPB and were subjected to brain temperature recording with the same plate temperature setting.

Following thermal plate preference tests or brain temperature recordings, the rats that had nanoinjections into the LPB additionally received bilateral injections at the same sites with 50 nl of 0.2% fluorescent microspheres (0.1 µm diameter; FluoSpheres, F8801; Invitrogen) to mark the sites. They were then subjected to transcardial perfusion and their brains were sliced to identify the location of the injection sites as described below.

### EEG recording

Following thermal plate preference tests, the rats that received saline or ibotenate injections into the VPM/PVL were anesthetized with 2.0% isoflurane in 100% O_2_ through a tracheal cannula. The trunk was shaved, a copper-constantan thermocouple to monitor skin temperature was taped onto the abdominal skin, and the trunk was wrapped with a plastic water jacket to cool and rewarm the skin. The rats were then positioned in a stereotaxic apparatus and a needle-type copper-constantan thermocouple (0.33 mm diameter; Physitemp) was inserted perpendicularly into the basal forebrain (2.2 mm anterior to bregma, 1.3 mm left to the midline, 7.0–7.5 mm ventral to the brain surface) to monitor brain temperature. Body core temperature was monitored with a thermocouple inserted into the rectum. All the thermocouples were connected to a thermocouple meter (TC-2000, Sable Systems, Las Vegas, NV, USA) for computer acquisition of the analogue signals. Bipolar electrodes for EEG recording were placed in the primary somatosensory cortex (0.5 and 2.5 mm posterior to bregma, 3.0 mm right to the midline and 1.0 mm ventral to the brain surface). During EEG recording, the isoflurane concentration was decreased to 0.8% and under this anesthetic condition, the animals never showed any movement except cooling-evoked shivering. EEG signals were amplified and filtered (×10,000, 1–30 Hz) with a CyberAmp 380 (Axon Instruments, Union City, CA, USA). Physiological variables were digitized and recorded to a computer hard disk using Spike 2 software (CED, Cambridge, UK).

Spike 2 software was also used to obtain a continuous measure (4-s bins) of EEG amplitude by calculating the root mean square amplitude of the variable (square root of the total power in the 0.5–2.5 Hz band for EEG) from the autospectra of sequential 4-s segments of these variables.

Following EEG recording, the rats were subjected to transcardial perfusion and their brains were processed to identify the lesioned areas as described below.

### Histochemistry

Rats were deeply anesthetized with chloral hydrate and transcardially perfused with 4% formaldehyde in 0.1 M phosphate buffer (pH 7.4). The brain was removed, postfixed in the fixative at 4 °C for 2–3 h, and then cryoprotected in a 30% sucrose solution overnight. The tissue was cut into 30-µm-thick frontal sections on a freezing microtome. The locations of the injections into the LPB were identified by detecting the fluorescent microspheres under an epifluorescence microscope.

To identify lesioned brain areas, brain sections of the rats that received injections into the VPM/VPL were processed for NeuN immunohistochemistry with our diaminobenzidine staining method^38^. Sections were incubated with an anti-NeuN mouse antibody (1 µg ml^−1^; MAB377; Merck Millipore, Billerica, MA, USA) overnight, and then with a biotinylated donkey antibody to mouse IgG (1:100; AP192B; Merck Millipore) for 1 h. The sections were further incubated with avidin-biotinylated peroxidase complex (ABC-Elite, 1:50; Vector, Burlingame, CA, USA) for 1 h. Bound peroxidase was visualized by incubating the sections with a solution containing 0.02% 3,3′-diaminobenzidine tetrahydrochloride (Sigma), 0.001% hydrogen peroxide and 50 mM Tris–HCl (pH 7.6). Some sections were separately stained with cresyl violet. All stained sections were mounted on aminosilane-coated glass slides and coverslipped.

### Anatomy and statistics

Most anatomical definitions of brain structures followed the brain atlas by Paxinos and Watson^39^. All data are presented as the means ± S.E.M. Statistic comparison analyses were performed using a paired or unpaired *t*-test or a two-way repeated measures ANOVA followed by the Bonferroni multiple comparisons test (Prism 6, GraphPad) as stated in the text and figure legends. All the statistic tests were two-sided. Statistical results with a *P* value of <0.05 were considered significant.

## References

[1] Morrison SF, Nakamura K (2011). Central neural pathways for thermoregulation. Front Biosci.

[2] Nakamura K (2011). Central circuitries for body temperature regulation and fever. Am J Physiol Regul Integr Comp Physiol.

[3] Terrien J, Perret M, Aujard F (2011). Behavioral thermoregulation in mammals: a review. Front Biosci.

[4] Cabanac M, Serres P (1976). Peripheral heat as a reward for heart rate response in the curarized rat. J Comp Physiol Psychol.

[5] Flouris AD (2011). Functional architecture of behavioural thermoregulation. Eur J Appl Physiol.

[6] Davis KD, Kwan CL, Crawley AP, Mikulis DJ (1998). Functional MRI study of thalamic and cortical activations evoked by cutaneous heat, cold, and tactile stimuli. J Neurophysiol.

[7] Becerra LR (1999). Human brain activation under controlled thermal stimulation and habituation to noxious heat: an fMRI study. Magn Reson Med.

[8] Kanosue K (2002). Brain activation during whole body cooling in humans studied with functional magnetic resonance imaging. Neurosci Lett.

[9] Craig AD, Bushnell MC, Zhang ET, Blomqvist A (1994). A thalamic nucleus specific for pain and temperature sensation. Nature.

[10] Craig AD (2002). How do you feel? Interoception: the sense of the physiological condition of the body. Nat Rev Neurosci.

[11] Nakamura K, Morrison SF (2008). A thermosensory pathway that controls body temperature. Nat Neurosci.

[12] Nakamura K, Morrison SF (2010). A thermosensory pathway mediating heat-defense responses. Proc Natl Acad Sci USA.

[13] Almeida MC, Vizin RCL, Carrettiero DC (2015). Current understanding on the neurophysiology of behavioral thermoregulation. Temperature.

[14] Gauriau C, Bernard JF (2004). A comparative reappraisal of projections from the superficial laminae of the dorsal horn in the rat: the forebrain. J Comp Neurol.

[15] Zhang X, Davidson S, Giesler GJ (2006). Thermally identified subgroups of marginal zone neurons project to distinct regions of the ventral posterior lateral nucleus in rats. J Neurosci.

[16] Gordon CJ (1990). Thermal biology of the laboratory rat. Physiol Behav.

[17] Almeida MC (2012). Pharmacological blockade of the cold receptor TRPM8 attenuates autonomic and behavioral cold defenses and decreases deep body temperature. J Neurosci.

[18] Cechetto DF, Standaert DG, Saper CB (1985). Spinal and trigeminal dorsal horn projections to the parabrachial nucleus in the rat. J Comp Neurol.

[19] Bernard JF, Dallel R, Raboisson P, Villanueva L, Le Bars D (1995). Organization of the efferent projections from the spinal cervical enlargement to the parabrachial area and periaqueductal gray: a PHA-L study in the rat. J Comp Neurol.

[20] Feil K, Herbert H (1995). Topographic organization of spinal and trigeminal somatosensory pathways to the rat parabrachial and Kölliker-Fuse nuclei. J Comp Neurol.

[21] Hylden JLK, Anton F, Nahin RL (1989). Spinal lamina I projection neurons in the rat: collateral innervation of parabrachial area and thalamus. Neuroscience.

[22] Li J (2006). Medullary dorsal horn neurons providing axons to both the parabrachial nucleus and thalamus. J Comp Neurol.

[23] Bratincsák A, Palkovits M (2004). Activation of brain areas in rat following warm and cold ambient exposure. Neuroscience.

[24] Kobayashi A, Osaka T (2003). Involvement of the parabrachial nucleus in thermogenesis induced by environmental cooling in the rat. Pflügers Arch.

[25] Nakamura K, Morrison SF (2008). Preoptic mechanism for cold-defensive responses to skin cooling. J Physiol.

[26] Lipton JM (1968). Effects of preoptic lesions on heat-escape responding and colonic temperature in the rat. Physiol Behav.

[27] Satinoff E, Rutstein J (1970). Behavioral thermoregulation in rats with anterior hypothalamic lesions. J Comp Physiol Psychol.

[28] Schulze G, Tetzner M, Topolinski H (1981). Operant thermoregulation of rats with anterior hypothalamic lesions. Naunyn Schmiedebergs Arch Pharmacol.

[29] Almeida MC, Steiner AA, Branco LGS, Romanovsky AA (2006). Neural substrate of cold-seeking behavior in endotoxin shock. PLoS One.

[30] Ray B, Mallick H, Kumar VM (2001). Role of the medial preoptic area in thermal preference of rats. Indian J Physiol Pharmacol.

[31] Tan CL (2016). Warm-sensitive neurons that control body temperature. Cell.

[32] Han S, Soleiman MT, Soden ME, Zweifel LS, Palmiter RD (2015). Elucidating an affective pain circuit that creates a threat memory. Cell.

[33] Sato M (2015). The lateral parabrachial nucleus is actively involved in the acquisition of fear memory in mice. Mol Brain.

[34] Miller RL (2012). Fos-activation of FoxP2 and Lmx1b neurons in the parabrachial nucleus evoked by hypotension and hypertension in conscious rats. Neuroscience.

[35] Geerling JC (2016). Genetic identity of thermosensory relay neurons in the lateral parabrachial nucleus. Am J Physiol Regul Integr Comp Physiol.

[36] Shin JW, Geerling JC, Stein MK, Miller RL, Loewy AD (2011). FoxP2 brainstem neurons project to sodium appetite regulatory sites. J Chem Neuroanat.

[37] Nakamura K (2002). The rostral raphe pallidus nucleus mediates pyrogenic transmission from the preoptic area. J Neurosci.

[38] Nakamura K (2000). Immunohistochemical localization of prostaglandin EP3 receptor in the rat nervous system. J Comp Neurol.

[39] Paxinos, G. & Watson, C. *The rat brain in stereotaxic coordinates*, *6th edn*. (Academic Press, 2007).

